# Supplementary material for: Responding to the Heat and Planning for the Future: An Interview-Based Inquiry of People with Schizophrenia Who Experienced the 2021 Heat Dome in Canada
Source: Int J Environ Res Public Health. 2024 Aug 21;21(8):1108. doi: 10.3390/ijerph21081108 (PMC11354195; doi:10.3390/ijerph21081108)
Supplement: Supplementary file 1 [file ijerph-21-01108-s001.zip › Supplementary Materials File S1.pdf]

An Interview-based Inquiry into the Experiences of People Diagnosed with Schizophrenia during the 2021 BC Heat Dome

Interview Guide for Those with Schizophrenia or Schizoaffective Disorder (and Their Family Members, Loved Ones or Caretakers) Who Survived the 2021 BC Heat Dome

Before the interview:

- Set up room, lay out snacks
- Test recorder
- Prepare 2 copies of the consent form (one for them and one for us to keep) and pen
- Have a copy of the resources sheet
- Go through the interview guide thoroughly so you have a full sense of what's being asked where, so you're able to comfortably jump around depending on how the conversation goes
- For interviews with family members/loved ones/caregivers, change the 'you' to 'they')

Make sure you are:

- Not squarely facing the participant
- Sitting closest to the door with one knee out rather than underneath the table
- Within reach of your panic button

To be paraphrased by interviewer:
I want to thank you for being here for this interview – your time is very valuable. The interview should take anywhere from 30 minutes to 60 minutes.

I first want to let you know that this device here is an audio-recorder. It's here to record our conversation so that I can remember what you said accurately. It's not recording yet – I'll only press the button to record once I have your consent before we start the interview.

I (Interviewer) am _____, and this is _____, and we're part of the team at UBC trying to better understand the experiences of people with schizophrenia or schizoaffective disorder during extreme heat events. We do have a particular focus on an event two years ago. Do you remember when it was really hot two summers ago in 2021? It was so hot that the heatwave got a special name of its own – the 2021 Western North America heat dome. [try to jog their memory back to 2021 summer] *We do have a focused interest in your experiences of that heatwave specifically, but it's ok if you'd rather speak about your experiences with heat more generally.*

[take mental note of whether they remember the 2021 heat dome or not]

I have some specific questions here that I'll ask, but I'm really interested in hearing your views, in your words, about your experiences with extreme heat. You're welcome at any point in the interview to add whatever you feel is important.

At any point during this interview, you can withdraw your participation by saying so (or by leaving the phone call / Zoom room). You can also choose not to answer any questions you don't feel comfortable answering, and afterwards, you can let me know if there's anything you said that you'd like to retract anything from the recording. I'd like to also make clear that this interview is not a form of treatment.

I will go ahead and start the recording once we've gone through the informed consent and after you've signed the consent form.

[Go through informed consent document together]
[Ask about their comfort with the recorder. Emphasize that only those on our research team will have access to this recording and we will anonymize all of your responses. If they're not comfortable, let them know you will take hand-written notes instead, which will not be shared with anyone but the research team, and will be shredded afterwards]

Does all of this sound alright? Do you have any questions before we get started?

Okay, I'll start the recorder.

[Start recording]

A) Introduction

It'd be great to get to know you a bit first. Could you tell me a bit about yourself? To the extent that you're comfortable, I'm interested to know who you are and how you've come to live/be here in [name of research site].

1. Where are you from, where did you grow up?
    - How old are you?
    - What is your marital status? (e.g. single, divorced, common-law, widowed)
2. What job(s) have you had before?
3. What do you like to do?
4. What events have led you here?

B) General

We want to know generally what your life was like for you during the time it was really hot two years ago, or any other heat waves.

5. Do you recall a time when it was really hot two years ago in 2021? What do you remember from that time?
    - It was around Canada Day, spanning the last week of June and first week of July in 2021. We were in the second summer of the COVID-19 pandemic. Do you remember a period where it was really hot, and other people were saying it was really hot? A time when it was hard to breathe the air?
6. Were you aware that a really intense heatwave was coming?
    - If so, how? (e.g., media, social networks, or any other specific targeted communication channels)
7. Do you recall where you (and your loved one) spent most of your time during the time that was really hot two years ago?
    - Where do you usually spend time when it's really hot?
8. What did your (and your loved one's) daily routine look like around that time? How did the heat wave affect it?
    - What does your usual summer day look like?

C) Medical Profile

To have a fuller understanding of your experiences with heat, I'd now like to ask you some questions about your health. Again, all of this information will be kept confidential. I'll first ask questions about your health now, and jog your memory back to two years ago.

9. How often do you/your loved one visit a physician or other medical professionals these days?
10. Are there other chronic physical or mental health conditions you/they currently have that you'd be open to sharing? *
    - How long have you had this/these condition(s)?
11. Do you/they use drugs or alcohol now?
    - If so, how often? **
    - Did you use drugs or alcohol two years ago when it was really hot?
12. Are you on any prescription meds now?
    - Would you be willing to share the prescription name?
    - Were you on the same meds two summers ago when it was really hot?
    - Can you recall if you were on any other meds two summers ago when it was really hot?
13. How do you feel when it's really hot outside compared to other days when it's not so hot?
    - Are there any activities you do or avoid when it's really hot outside?
    - Do you recall what you did when it was really hot two years ago?
14. Are you able to move around on your own generally without any assistance?
    - Are there ever times you're stuck in bed? What leads to those times?
    - Are there ever times you require help to walk? Like using a cane/walker, or a wheelchair?
15. How do you usually dress? What do you like to wear?
    - Do you ever change the way you dress because of how hot it is? If so, how? What does that look like?
    - Does how you/they dress change throughout the year? If so, how so?

D) Living Situation, Built Environment, Social Vulnerability and Connections

I'd like to better understand what your living environment was like before you got here, leading back to two years ago.

16. Where were you/they living before you arrived here?
    - What neighbourhood did you live in?
    - How long had you lived there for?
    - Did you live there two summers ago when it was really hot outside?
17. What type of building was your home in? (A single detached home? An apartment?)
    - If an apartment, how many floors are there in the building and which floor did you live on?
    - Neighbourhood, type of home, building (what floor of how many floors)?
    - Did you know your neighbours? Did they know you? Did you say hi or ever hang out?
    - Did you live there with anyone?
18. What is your social circle like? Do you have close family and friends? Part of any common interest groups?
    - How do you keep in touch with friends or family or other people?
        - By phone? In-person visits? How often?
    - Did anyone come to visit you while you lived there? Friends or family?

- Do you recall talking to anyone in person or on the phone or text when it was really hot two summers ago?

E) Mitigation Measures

Now I'd like to understand your access to resources to keep you cool during the heat

19. How do you usually keep cool when it's really hot outside?
    - Did you do this two summers ago too?
20. Do you recall having an air conditioner or fan at home?
    - Did you use it during the time it was really hot two summers ago?
    - Did you get to control the A/C or fan yourself or did someone else?
21. Did you have a thermostat at home? Do you remember seeing what temperature it was?
22. Did you know about publicly available spaces to cool off, called 'cooling centres'?
    - If so, did you go to them? How did you find out about them?
    - Or, do you recall why you didn't or couldn't go to them?
    - Do you recall others telling you about any places to go to cool off?
23. Did you have a steady income before you arrived here?
    - Was this the case two summers ago when it was really hot?
    - Did you buy anything to keep you cool?

F) Looking Ahead

24. What do you find is helpful to make you feel cool and safe when it's hot?
    - If you had a magic wand, what would you wish for to keep you cool and safe when it's hot outside?
25. Back to two summers ago, is there anything you wish had gone differently when it was really hot that summer?
26. Is there anything else you'd like to add? Or anything else I didn't touch upon you'd like to say?

[Turn off recorder now]

Thank you! That was great. [any personal reflection on the interview the interviewer may wish to share]

Here is your gift card / cash to thank you for your time. If you have any questions afterwards, please let your PCC know or talk to any one of us as you'll see us around a times a week for the next little while.

* Physiologic evidence has shown that various chronic conditions, such as cardiovascular disease, lung disease, diabetes, hypertension, among others, impair the body's ability to thermoregulate in the heat. Further, in addition to chronic conditions which physiologically impair thermoregulation, other conditions may impact mobility and cognition, including other mental health conditions (e.g., mood and anxiety disorders, and depression), which could affect a person's ability to recognize overheating and to take protective action. For example, conditions like osteoarthritis reduce mobility which could impede an individual's capacity to attend a cooling centre, and conditions which cause cognitive decline like dementia may potentially impact a person's ability to understand or to respond to extreme heat, or to self-rescue by hydrating, or finding other relief. Therefore, we will be asking participants to voluntarily share other chronic physical or mental health conditions they may have to explore potential compounding risk and vulnerability to the heat.

**Individuals who use substances, including toxic drugs and alcohol, are at an increased risk of negative health outcomes during extreme heat events. Further, there is known correlation between diagnosis of substance use disorder and heat related morbidity and mortality. Therefore, we will be asking participants to disclose

**Individuals that use substances, including toxic drugs and alcohol, are at an increased risk for negative health outcomes during extreme heat events. This risk is due to both physiologic impairments (e.g., sweating response, vasodilation), and impairments to behavioural response initiation (i.e., ability to detect heat and mitigate risk). Further, there is a known association between diagnosis of substance use disorder and heat-related morbidity and mortality. Therefore, participants will be asked to optionally share if they use substances to help elucidate any additional risk or vulnerability to the heat.
