# Supplementary material for: Responding to the Heat and Planning for the Future: An Interview-Based Inquiry of People with Schizophrenia Who Experienced the 2021 Heat Dome in Canada
Source: Int J Environ Res Public Health. 2024 Aug 21;21(8):1108. doi: 10.3390/ijerph21081108 (PMC11354195; doi:10.3390/ijerph21081108)
Supplement: Supplementary file 1 [file ijerph-21-01108-s001.zip › Supplementary Materials File S2.pdf]

Responding to the Heat and Planning for the Future: An Interview-Based Inquiry of People with Schizophrenia Who Experienced the 2021 Heat Dome in Canada

Supplement B: Codebook

Themes	Concepts		Sub-Concepts		Interviewee Example (Excerpt)
	Concept	Definition	Concept	Definition	
**Personal Reflections on the Impact of Extreme Heat**	**Impacts on Cognitive Capacity, Emotional and Mental Stability**	Any content related to mental processes involved in the acquisition of knowledge, manipulation of information and reasoning, such as perception, memory, learning, attention, decision making and language, and any content related to changes in mental state as a result of the heat.	Sleep	Any content related to causing disruption to an individual's sleep behaviours, including quality and quantity of sleep.	*"During that time, no air conditioning and it was really hot. And also my sleep quality has deteriorated because of that heat too…Because that because I was so stressed out already my mind was getting was hearing like, like, stressed out not getting enough sleep. That I started getting psychotic again." (C03 – 23; male; housed; no substance use; not medicated)*
			Disorientation	Any content related to being in a state of confusion or being disoriented.	*"And kind of maybe even a bit disoriented a little bit to some degree, like not heatstroke, but, like out of sorts a little bit from it" (A12 – Male, 42, Mobile home, Substances - Cough syrup, vape, dill, coke, acid, Medicated - Olanzapine, Concerta)*
			Lethargy	Any content related to lacking energy or feeling sluggish.	*"I was homeless. Tough to be in the heat and uh I'd question myself like him in this like taking like life for me like it's so hot to draining that to life away from me, you're us you're uh from the future and the stuff. Yeah, drains you from all that energy from being in the heat. Drink water is important because dehydrated and stuff. Gotta be smart with it I guess (A14 – Male, 33, unhoused, substance use- heroin, cocaine, crystal meth, medication - Suboxone, tylenol, haldol)*
			Mental capacity	Any hindrance of mental capacity or capabilities.	*"I was like, forgetting how to even though I've written tons of essays, I was like, forgetting how to…write. And it was, and I don't do everything properly" (C02 – Female, age unknown, no substance use, medication - sulfasalazine, seroquel, clonazepam, emtec, pantoprazole, flurbiprofen, simponi,, independently housed)*
			Anxiety	Any anxious feelings or thoughts related towards the heat dome or other events.	*"Anxious. anxious. I feel really anxious when I'm hot." (C05 – 57, female, independently housed, medicated - clozapine)*
			Adhering to treatment	External or internal factors influencing one's ability to manage treatment.	*LY: "Right. Okay. So it's hard to be on top of your treatment when it's really hot." A01: "Yeah." (A01 – age not specified; male; unhoused; substance use: heroin, crack, speed; medicated: depot shot, methadone)*
			Emotional toll	Negative emotions placing external pressures on the participant, causing decreased mental states.	*"I only learned through the grapevine that SO many people, SO many people that I knew from the clubhouse died." (C02 – age unknown, Female, independently housed, medication - sulfasalazine, seroquel, clonazepam, emtec, pantoprazole, flurbiprofen, simponi)*
			Aggression and violence	Angry and/or violent actions and/or thoughts relating to the heat dome or caused by heat-related factors.	*"(being) irritable and wanting to fight" (A14 – 33, Male, unhoused, substance use- heroin, cocaine, crystal meth, medication - Suboxone, tylenol, Haldol)*
			Stress	Physical or emotional symptoms of stress, caused by heat, schizophrenia, or personal factors.	*"Like when I'm when I'm upset, like, if I'm mad, like I had to learn to really talk. Like, stay calm, you know, or if I'm stressed out, like, if something is really stressing me out and he, he picks up on that, then the voices will get a bit louder, or he has to speak publicly." (D01 – 42, Female, independently housed with spouse, Substances – Alcohol, Medicated - Clozapine)*
	**Worsening of Symptoms Associated with Schizophrenia**	Any content related to schizophrenia symptoms that were aggravated due to the heat.	Trigger for psychosis	Any content that describes an individual experiencing aspects of psychosis being a trigger or increasing in the heat.	*"And when I was outside in the heat, I'd be too tired to have my schizophrenia go off. But when I was back inside, it would be going crazy as soon as the heat went away." (A13- 24, male, independently housed, meth, fentanyl, benzos, medicated - olanzapine- 20 milligrams, Kadian - 1900 milligrams, champix, cigarettes)*
			Trigger for hallucinations	Any content that describes an individual experiencing aspects of hallucinations being a trigger or increasing in the heat.	*"Heat causes more: "Mental disturbances, just delusional thinking and yeah, hallucinations." (C03 – 23; male; housed; no substance use; not medicated)*

			Discontinued medication use	Temporarily not using medication in the heat.	*"I discontinued it [the prescription drugs] because it was just so fucking hot." (A01- male, homeless, Heroin, crack, speed, medicated - Depot shot (monthly), methadone; Heroin, crack, speed)*
			Dehydration	Descriptions of not having enough water.	*"It would just like, the sweating and the malnutrition of it, and not having water. And I guess it just, seized my brain. You know, like, no water with the brain, you know" (A01- male, homeless, Heroin, crack, speed, medicated - Depot shot (monthly), methadone; Heroin, crack, speed)*
	**Physical impacts of Heat**	Any content related to the impairment of normal bodily functioning due to the heat.	Challenges with Breathing	Any content that describes breathing difficulties.	*"I remember the air conditioning couldn't keep up with the heat. And I remember we decided to go for a walk when it was like 41 degrees outside. And I remember being able to not really breathe very easily. And I felt really hot and very exhausted." (A12 – Male, 42, Mobile home, Substances - Cough syrup, vape, dill, coke, acid, Medicated - Olanzapine, Concerta)*
			Sweating	Any content that describes sweating or perspiring.	*"Oh, year. I sweat more I- I feel it causes as such it's like, at the beginning of slight sensation of stress in the body because of sweating more and feeling that moisture on your. Skin that really hot feeling. And then it. Year, it also makes me more anxious, more and more anxiety, too. So like that, that can contribute to like my mental stability, too. Right." (C03- 23, male, independently housed, medicated - risperidone, anti-depressant, anti-psychotic)*
			Heat-related illness	Any content that describes illness or impaired health state due to heat.	*"You ever been outside where it's so hot and you feel dizzy? Like you just don't feel, like, right? It's not even like, any problem or anything like, you, know, it's not your fault. It's the outside's fault and you can't stop being dizzy and you just don't feel like you're even in reality anymore. That's kind of what it was like." (A10 – Male, 31, Duplex Substances - Cannabis, Liquor, Cocaine, Medicated - Lamotrigine, Escitalopram, Abilify)*
	**Impacts on Everyday Life**	Any content related to heat affecting participants' lifestyle.	Work	Any content related to heat affecting participants' work.	*"I actually can't go – I lose a lot of work because of that, because I can't commute because the buses don't have air conditioning…it's terrible. And I literally get sick on it." (D01 – 42; female; Independently housed with spouse; substance use: alcohol; medicated: Clozapine)*
			Hobbies	Any content related to heat affecting participants' hobbies.	*"I stopped running during the heat because it was just too hot." (C08 – 40; male; housed; no substance use; medicated: Olanzapine)*
			School	Any content related to heat affecting participants' schooling and education.	*"And I was like, forgetting how to even though I've written tons of essays, I was like, forgetting how to... [write]. And it was, and I didn't do everything properly…. but I was forgetting how to construct an essay and, um, I guess I, there was just a lot of fog, and I'm just, I'm not back to where, I am not back to the ability I had before the heat dome." (C02 – age not specified; female; housed; no substance use; medicated: sulfasalazine, Seroquel, clonazepam)*
			Other	Any content related to heat affecting participant's lifestyle in aspects other than work, hobbies, and school.	*"I couldn't cook. I remember that. I could not cook. Because if you turned the elements on or the stove on at all, it's way too hot. We had to order Domino's like every day." (A10 – 31; male; housed; substance use: cannabis, liquor, cocaine; medicated: Lamotrigine, Escitalopram, Abilify)*
**Cooling Strategies Employed to Mitigate the Heat**	**Individual-Level Cooling Strategies**	Any content related to the use of individual-level cooling strategies to cool the body.	Electric Fans	Any content related to people using electric fans (e.g., pedestal, ceiling) to enhance cooling.	*"And then we also have fans in the windows. So the window fans. So we would have. Yeah, we would bring it in the nighttime, air it in, and then send it out during the day. So we did that. All through that time. And then we had like tower fans. We have two tower fans as well." (Participant C08)*
			Self-Dousing	Any content related to people self-dousing or wetting themselves with water and/or ice.	*"And I ended up surviving just with the, the one uh powered fan that I have. But I also learned about new tricks. Like you can sleep with your cold socks or wet socks, and that will help regulate your body... It's like, you know, like wet clothes or damp clothes. kind of help regulate your body." (Participant C04)*
			Water Immersion	Any content related to people submerging in the bathtub, shower, pool or other body of water for cooling purposes.	*"My husband would put me in the shower and run cold water over me. And so that was he would have done that three times a day." (Participant C02)*
			Consuming Cool Drinks and Food	Any content related to people consuming specific food and/or drinks for cooling.	*"Drinking water, hydrating a lot. Drinking lots of water… I remember hydrating a lot, drinking a lot of water. Yeah." (Participant A16)*
			Optimizing Clothing	Any content related to people removing or selecting specific clothing items for enhanced cooling, such as light-weight, light-colour options.	*"Oh, usually short…Or actually, if it were possible, like, like, the summer, I don't know what they call. Something's made out of cotton or not cotton or linen. Like linen cotton. I don't know the differences. Just something just like doesn't make me feel hot. Lightweight clothing. And I would wear shorts. I would wear*

					shorts outside. Yeah, shorts. Like, like, like those short pants. You know, shorts. Like just on your knees." (Participant C03)
			Reducing Time Outdoors	Any content related to people modifying their daily routines to reduce direct exposure to the heat or sunlight.	*"But I would generally stay outside late at night since it cooled off and be inside during the day. So I actually switched my sleeping schedule around so I would be up at night and asleep during the heat."* (Participant A04)
			Reducing Activity Level	Any content related to people reducing their energy expenditure during the heat.	*"I avoid going outside and walking, walking when I don't need to, when it's hot outside, obviously."* (Participant C03)
			Hair cut	Any content related to people modifying their hair to reduce heat stress.	*"I was homeless. Tough to be in the heat… That's why I got this haircut, it's because of the heat."* (Participant A14)
			Lying on the Floor	Any content related to people lying on the floor to enhance cooling.	*"I do remember, like, having to go and have a cold shower and, in my apartment, like, lay down on the floor. Because the heat rises, right?... I was able to manage it by getting down closer to the floor and regulating it with fans."* (Participant C06)
			Limiting Heat-Generating Appliance Use	Any content related to people trying to reduce heat exposure by limiting appliance use, such as the oven.	*"Turn off your oven. Like if you don't have to cool like with a big oven, you have a stovetop oven or as countertop use that instead."* (Participant C04)
	**Building-Level Cooling Strategies**	Any content related to the use of building-level cooling strategies to cool the space.	Mechanical Cooling	Any content related to having or activating mechanical cooling options (e.g., a/c, heat pumps, etc.)	*"It didn't really affect me too much. Because it was during COVID. And I was kind of isolated extremely. My place was very well air-conditioned. So I spent the entire summer, like right inside. So the heat didn't really affect me that year."* (Participant A07)
			Natural Ventilation	Any content related to the use of natural ventilation methods, such as windows and doors to create cross drafts.	*"Yeah, actually, I do remember; what we did was we would leave the windows open at night and shut them in the day, including the balcony door; we'd have that open at night and shut it in a day. My only concern with that was like if it was a security risk, but we just felt like the risk was outweighed by the benefit of having a cooler house."* (Participant C08)
			Window Shading	Any content related to the use of window shading, such as blinds, curtains, shutters to reduce sun from entering the home.	*"Like just being cool. And making sure I'm in the shade and making sure that the draw the blinds are the… Yes. The curtains are closed… Yeah. And I always like I always keep the door open all the time. All the time, all the time, and the screens shut."* (Participant C06)
			Wetting Surfaces	Any content related to using water to lower the surface temperature of the home or surroundings.	*"During the heat, was pour water on the balcony to see if it would cool it."* (Participant C02)
	**Other Cooling Strategies Used in Alternative Living Environments**	Any content related to the use of alternative cooling strategies to achieve cooling in alternative living environments.	Seek Shaded Areas	Any content related to seeking shaded areas outside when indoor access was not available.	*"Um, I just tried to find, like, a shady tree or something. I lived in the, in the parks, um, underneath trees and stuff. So you know, I would just stay in the shade."* (Participant A02)
			Locate Cool Sleeping Areas	Any content related to finding locations to sleep outdoors that offered cooler surroundings.	*"You have to sleep in the shade, like under a tree or something. It's just too hot."* (Participant B04)
			Access Cool Public Spaces	Any content related to accessing cool public spaces such as malls and libraries.	*"Staying indoors was generally one of the greater methods cause it was air-conditioned at a couple of other places that I would go to. They had a place called Anchors, which is a use, how do you describe it, the substance use kind of clinic where you go you get equipment for using or just go inside for a bit and talk to them, get drinks."* (Participant A04)
			Use drugs	Any content related to using non-prescribed drugs to achieve a cooling effect.	*"I ate cold foods. And sometimes I'd grab ice and put it on my head if I needed it. But otherwise, I just used drugs, and the heat just kind of didn't seem as bad if I was high"*(Participant C04).
**Perspectives and Suggestions for Future Heat Protection**	**Structural and Societal Mitigations and Adaptations**	Participants' wishes and recommendations for interventions at the structural and societal level that would improve	Outreach Services and Foot Patrol	Any content related to the need for foot patrol and outreach services in dense urban areas to help identify those experiencing a heat-related illness or in need of support.	*"If there would have been some sort of reaching out program to everyone. You know, maybe they didn't want to or when we could have signed up for this somehow broadcasts like, you know, hey, this is a wellness program. It brand new because of the hope because of COVID. And the heat dome, it's a unique situation we're in, you know, our services are free of charge. Give us a call, we'll call you. And what is it that you need? Like what does it that you specifically feel like you need for this period?" (Participant C04)*

		health outcomes for individuals with schizophrenia during heat events.	Training for Healthcare Providers	Any content related to the need for training for health care providers to help better support those with schizophrenia in advance of an extreme heat event.	*"And almost every healthcare student, and it's not only nurses, it's also medical professionals, OTs, social workers, kinesiology, all of that. They get no training in mental health, they get so much training and so many other things. They have no idea about mental illness, when I talk to them about mental illness, serious chronic chronic mental illness, they have no clue. They have no clue. They don't know. So it's an- and believe me, there are hardly anybody I met who's high functioning like me with my condition." (Participant C05)*
			Peer Support and Social Interaction	Examples supporting benefit of social connection/ resources.	*"I think it's just an isolating illness. Like even being here, The nurses are like, are you hearing voices, what are they saying? And then that's it, you know. So I think social interaction and social dynamics and stuff because it's such an isolating illness, you know. It's like not fitting in in that way is like a major problem. I think that's way more important than the heat, is um, social networking somehow. So I got something I can do in the future even be a peer support worker, something like that (Participant B07)*
			Enhanced Alerting	Any content related to participants expressing the need for better alert systems for extreme heat events in the future.	*"Right, right. Yeah. So no, they really do need to educate on that and, and posters, posters, posters, in every neighborhood as to where the closest is to that neighborhood of cooling stations, or even like outdoor shower, like cooling cooling spots when a misting spot. And more outreach, like really? And this Yeah, totally coming from a job perspective. But I've always we've always needed more outreach. But summer, for sure. I mean, that's hard on the workers as well." (Participant D01)*
			Representation on Committees	Any content related to participants expressing a desire to be present and have representation on committees.	*"Why are we not listening to the people that have gone through it? They have so much to share"* (Participant *C05*).
			Home Check-In Services	Any content related to participants expressing a desire for check-in services to come to their home during extreme heat events.	*"If there was someone that was like, you know, randomly calling people, it's like, you know, hey, you know, communicating. Hi, my name is so and so from so and so. How are you doing, you know, and engage that person to say, Well, I'm stuck at home, and I can't get my groceries. Yeah and there's a service that could have been, you know, help with that person. Or, I'm always just too hot food, it's too hot in my unit. And then you know, bringing in fans and ice chests or something like that, or something to really engage with someone, as an individual person." (Participant C04)*
			Public Awareness of Schizophrenia	Any content relating to a participant addressing the discrimination they have seen or experienced surrounding mental illnesses, or changes they believe need to be made societally.	*"So it's, it's really hard dealing, dealing. Having a mental illness and dealing with the prejudices in society is really hard. And I was surprised to see this study. Because I was surprised anyone cared [about people with schizophrenia]." (Participant C02)*
			Community Cooling Resources	Any content regarding wishing for more water access	*"I was living outside and, you know, going from watering hole to watering hole basically, like, trying to keep hydrated. And you know, keep my hygiene, my hygiene. You know, good at the same time. Trying to find open showers and stuff like that, you know. And um, trying to keep cool" (Participant A01)*
			Home Cooling Evaluation Services	Any content related to the desire for an at-home cooling assessment.	*"I think it would be nice to have access to someone to evaluate the place that we live in. Because, like some sort of like, heating cooling expert to see what we need. It's hard for me, it's hard for me to imagine, like, it's hard for me to imagine what I know I need to be cooler." (Participant C02)*
	**Individual Behaviour-Oriented Mitigations and Adaptations**	Participants' wishes and recommendations for interventions at the individual level that would improve health outcomes for individuals with schizophrenia during heat events.	Personal Resources	Any content where the participant discusses material resources that would aid their current lifestyle or heat mitigation	*"A house with... I'd like a small house with um, what do you call it, with the kind of air conditioning that goes through the air ducts?" (Participant C02)*
			Education	Any content where a participant expresses a desire for resources about heat mitigation or extreme heat events.	*"I would say, most of the people that could benefit from maybe more social resources for navigating your own personal wellness goal [...] I don't know how that would have worked. But like, if there was someone that was like, you know, randomly calling people, it's like, you know, hey, you know, communicating. Hi, my name is so and so from so and so. How are you doing, you know, and engage that person to say, Well, I'm stuck at home, and I can't get my groceries. Yeah and there's a service that could have been, you know, help with that person. Or, I'm always just too hot food, it's too hot in my unit. And then you know, bringing in fans and ice chests or something like that, or something to really engage with someone, as an individual person." (Participant C04)*

	**Built Environment Oriented Mitigations and Adaptations**	Participants' wishes and recommendations for interventions regarding the built environment that would improve health outcomes for individuals with schizophrenia during heat events.	Home Modifications	Desire for window film	*"Yeah, I mean, I can see why they wouldn't want the shiny silver ones because it can reflect and blind people temporarily, but those the film that you can put on that, um, that, you know, if we were allowed to do that, that would make a difference. And also, as our building told us that we weren't allowed..." (Participant C02)*
			Green Space	Any content related to participants' expressing the need for more shaded areas to better manage extreme heat events	*"So I don't really feel comfortable on going to crowded places that much right now. Like the beach or the park and you know, I mean clothes, booming, there's a whole bunch of new people there. And so I I'll go a couple times during the day and sort of investigate things and then come back to my, my room that I rent." (Participant B07)*

Note: All codes are considered descriptive and were developed inductively.
