# Supplementary material for: Responding to the Heat and Planning for the Future: An Interview-Based Inquiry of People with Schizophrenia Who Experienced the 2021 Heat Dome in Canada
Source: Int J Environ Res Public Health. 2024 Aug 21;21(8):1108. doi: 10.3390/ijerph21081108 (PMC11354195; doi:10.3390/ijerph21081108)
Supplement: Supplementary file 1 [file ijerph-21-01108-s001.zip › Supplementary Materials File S3.pdf]

An Interview-based Inquiry into the Experiences of People Diagnosed with Schizophrenia during the 2021 Heat Dome

Demographic, Substance Use, and Medication Use Data

Table s1. Self-reported demographic characteristics of participants.

Characteristics	Total *(n=35)*
**Sex**	
Female	12
Male	23
**Age (years)**	
20-29	8
30-39	9
40-49	5
50-59	4
60-69	1
70-79	2
80+	1
Unknown	5
**Marital Status**	
Single	20
Married/Common Law	3
Divorced	5
Widowed	1
Did not Disclose	6
**Sheltered During Heat Dome**	
Sheltered	28
Unsheltered	6
Did not Disclose	1

Table s2. Self-reported non-prescribed substance use of self-identified female and male participants living with schizophrenia or schizoaffective disorder during the 2021 Western Heat Dome (n = 35).

**Non-Prescribed Substances**	**Total** *n*	**Female** *n*	**Male** *n*
**Total**	36	12	23
Meth/ Speed	15	5	10
Alcohol	11	2	8
Cocaine/ Crack	9	3	6
Weed/ Cannabis/ Marijuana	8	2	6
Heroin	6	1	5
None	6	4	2
Fentanyl	3	1	2
Unknown	3	0	3
Acid/ LSD	2	1	1
Benzodiazepines	2	0	2
Cigarettes	2	1	1
Ecstasy/ Molly	2	1	1
Cough syrup	1	0	1
Dill	1	0	1
Hydromorphone	1	0	1
Ketamine	1	1	0
Morphine	1	0	1
Mushrooms	1	1	0
NAS	1	1	0
Not disclosed	1	1	0
Oxycodone	1	0	1
Percocet	1	0	1
Tylenol-3's	1	0	1
Vape	1	0	1

Note: ‘Unknown’ refers to individuals that disclosed drug use but did not indicate the type of drug(s).

Table s3. Comparing self-reported prescription medication use of self-identified female and male participants living with schizophrenia or schizoaffective disorder, during the 2021 Western Heat Dome compared to the present day.

Prescribed Medication	Female *n*		Male *n*	
	During Heat Dome	Present	During Heat Dome	Present
Specific Medications				
Aripiprazole	2	2	3	2
Acamprosate calcium	0	0	0	1
Amphetamine/dextroamphetamine	1	1	0	0
Benztropine mesylate	1	1	0	0
Buspirone hydrochloride	0	0	1	1
Clonazepam	2	3	1	0
Clozapine	3	2	1	7
Methylphenidate extended-release	0	0	1	0
Desmopressin acetate	0	0	0	1
Dextroamphetamine	0	0	1	1
Acetaminophen and Codeine	0	1	0	0
Escitalopram oxalate	0	0	1	0
Flurbiprofen	0	1	0	0
Gabapentin	0	0	0	1
Haloperidol	0	0	1	1
Hydroxyzine hydrochloride or Hydroxyzine pamoate	1	1	0	0
Lamotrigine	0	0	1	0
Lithium carbonate or Lithium citrate	0	0	1	0
Loxapine	1	1	1	1
Metformin hydrochloride	0	0	0	1
Methadone hydrochloride	0	0	2	2
Nadolol	0	0	0	1
Naltrexone	0	0	0	1
Olanzapine	0	0	4	1

Paliperidone	0	0	4	4
Pantoprazole sodium	0	1	0	0
Quetiapine fumarate	1	1	0	0
Risperidone	0	1	0	1
Quetiapine fumarate	1	1	0	0
Golimumab	0	1	0	0
Buprenorphine and Naloxone	0	1	1	2
Sulfasalazine	1	1	0	0
Topiramate	0	1	0	0
Acetaminophen	0	0	1	1
**Round pearl?**	0	0	0	1
Sertraline hydrochloride	0	0	0	1
Valproic acid	0	0	0	1
Morphine sulfate extended-release	0	0	0	2
Cholecalciferol	0	0	0	1
Ferrous sulfate, Ferrous gluconate	0	0	0	2
Methotrimeprazine	0	0	0	1
Phenelzine sulfate	0	0	0	1
Varenicline	0	0	0	1
Ascorbic acid	0	0	0	1
Ambroxol	0	0	0	1
Lorazepam	0	1	0	0
Trazodone hydrochloride	0	1	0	1
General Medication Classifications				
Anti-depressant	0	0	0	1
Anti-psychotic	0	0	0	1
Sz meds	0	1	0	0
Unknown	6	3	8	5

Note: ‘Unknown’ refers to individuals that disclosed drug use but did not indicate the type of drug(s).
